# The Dectin-2 family of C-type lectins in immunity and homeostasis

**DOI:** 10.1016/j.cyto.2009.07.010

**Published:** 2009-10

**Authors:** Lisa M. Graham, Gordon D. Brown

**Affiliations:** a Wernher & Beit Building South, Institute of Infectious Disease and Molecular Medicine, University of Cape Town, Observatory, 7925, Cape Town, South Africa; b Section of Immunity and Infection, Institute of Medical Sciences, Ashgrove Road West, University of Aberdeen, Aberdeen AB25 2ZD, UK

**Keywords:** C-type lectin, Carbohydrate recognition domain, ITAM, ITIM

## Abstract

C-type lectins are a diverse family of proteins which recognize a wide range of ligands. This review focuses on the Dectin-2 family of C-type lectins that includes Dectin-2, BDCA-2, DCIR, DCAR, Clecsf8 and Mincle whose genes are clustered in the telomeric region of the NK-gene cluster on mouse chromosome 6 and human chromosome 12. These type II receptors are expressed on myeloid and non-myeloid cells and contain a single extracellular carbohydrate recognition domain and have diverse functions in both immunity and homeostasis. DCIR is the only member of the family which contains a cytoplasmic signalling motif and has been shown to act as an inhibitory receptor, while BDCA-2, Dectin-2, DCAR and Mincle all associate with FcRγ chain to induce cellular activation, including phagocytosis and cytokine production. Dectin-2 and Mincle have been shown to act as pattern recognition receptors for fungi, while DCIR acts as an attachment factor for HIV. In addition to pathogen recognition, DCIR has been shown to be pivotal in preventing autoimmune disease by controlling dendritic cell proliferation, whereas Mincle recognizes a nuclear protein released by necrotic cells. Here we review each of these receptors in detail describing their expression, ligand recognition, signalling and known physiological functions.

## 1 Introduction

To execute both immune and non-immune functions, leukocytes interact with various cell types, both directly and through soluble factors. These interactions are controlled by an array of receptors which are essential for diverse but specific functions, including leukocyte development, differentiation, migration, proliferation, maturation and survival, as well as for discrimination between non-threatening self-antigens and foreign matter. Many receptors also have the ability to distinguish between healthy and abnormal self, such as virally infected, tumorous or apoptotic/necrotic cells. The ligands for these receptors vary greatly, ranging from endogenous molecules to specific molecular structures found in microbes called pathogen associated molecular patterns (PAMPs) [1]. PAMPs are not found in the host, enabling discrimination between self and non-self and are essential for the microbe’s survival, limiting the possibility of escape through mutation. Although termed ‘pathogen associated’, it is important to note that PAMPs are also found in nonpathogenic microbes [2]. The receptors recognizing these structures are germ-lined encoded and have been termed pattern recognition receptors (PRRs) [3].

A family of receptors of particular interest are the C-type lectins; a diverse group of proteins which were originally defined by their ability to recognize carbohydrate structures [4]. C-type lectins execute both immune and non-immune functions. While some recognize endogenous ligands to facilitate adhesion between cells, adhesion of cells to extracellular matrix and other non-enzymatic functions, others may act as PRRs.

C-type lectins consist of a distinct protein fold, termed the carbohydrate recognition domain (CRD), which is generated through disulphide bridges between conserved cysteine residues. This family has been divided into 17 groups based on the organization of their CRDs, and can be functionally defined as either classical or non-classical [5,6]. Classical C-type lectins contain conserved residues in their CRDs which are responsible for forming Ca^2+^ binding sites and also generally contain conserved motifs which typically bind carbohydrate ligands, such as the EPN amino acid triplet which binds mannose-type carbohydrates or the QPD triplet which binds galactose-type carbohydrates. Non-classical C-type lectins or lectin-*like* receptors generally do not contain these residues and are more likely to, but do not necessarily, bind non-carbohydrate ligands, such as those encoded by the Natural killer-gene complex (NKC) which control cellular activation by recognition of MHC class I molecules [5,7].

Upon ligand recognition, C-type lectins can induce a variety of cellular responses, and can be further functionally divided into those that inhibit or those that induce cellular activation. In general, inhibitory receptors contain a consensus immunoreceptor tyrosine-based inhibitory motif (ITIM) in their cytoplasmic domains, while activation receptors either contain an immunoreceptor tyrosine-based activation motif (ITAM), or associate with signalling adaptor molecules such as DAP12, DAP10 or FcRγ chain. In myeloid cells, signalling through an ITAM-bearing receptor results in activation of Src homology 2 (SH2) domain-containing protein tyrosine kinases (e.g. Syk) which leads to various cellular outcomes, such as induction of phagocytosis, the respiratory burst and the production of cytokines and chemokines [8]. Upon phosphorylation of ITIM-bearing receptors, SH2-containing phosphatases are recruited which dephosphorylate the tyrosines of activation kinases, thereby downmodulating cellular activation [9]. Recently however, it has become apparent that this dichotomy is not always clear cut, as some ITAMs have been shown to mediate cellular inhibition while ITIMs induced activation [10,11]. None the less, it is generally accepted that pair(s) of immunoreceptors on cells maintain the balance between cellular activation and inhibition and defects in this control have often been associated with excessive inflammation, autoreactivity and disease [12–14].

**Fig. 1:**
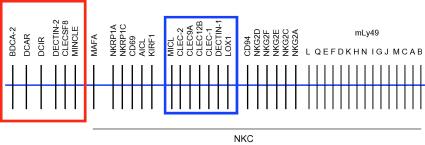
The Dectin-2 family genes form a cluster in the telomeric region of the NKC. The Dectin-2 gene family includes BDCA-2, DCAR, DCIR, Dectin-2, Clecsf8 and Mincle, and form a cluster (red square) in the telomeric region of the NKC, close to the Dectin-1 cluster (blue square), on mouse chromosome 6 and human chromosome 12.

To date, particular interest has been paid to the C-type lectins encoded in the NKC, situated on mouse chromosome 6 and the syntenic region on human chromosome 12p13 [7]. Although many receptors of the NKC are expressed primarily by NK and T cells, a growing number have been found to be expressed on myeloid cells [15]. In contrast to the NK and T cell specific receptors which function mostly in detection of tumorous or virally infected cells, largely by means of MHC class I recognition, the myeloid expressed receptors seem to have a more diverse repertoire of ligands and cellular functions, including pathogen recognition and maintenance of homeostasis [7]. The Dectin-1 cluster of receptors is one such example, which includes Dectin-1, lectin-like oxidized low-density lipoprotein receptor-1 (LOX-1), C-type lectin-like receptor-1 (CLEC-1), CLEC-2, CLEC12B, CLEC9A and myeloid inhibitory C-type lectin-like receptor (MICL) and form part of the Group V C-type lectin-like receptors [16]. These receptors have similar structural features but recognize diverse endogenous and exogenous ligands. CLEC-2 for example, recognizes the endogenous ligand podoplanin, as well as the snake venom toxin rhodocytin [17–20]. Dectin-1 has been shown to specifically recognize fungal (1,3)-linked β-glucans, while CLEC9A recognizes a ligand in necrotic cells [21,22]. Another cluster of receptors gaining interest is the Dectin-2 family of C-type lectins. These receptors are clustered in the telomeric region of the NKC, in close proximity to the Dectin-1 family (Fig. 1) and also appear to have diverse functions in both immunity and homeostasis.

## 2 The Dectin-2 family of C-type lectins: an overview

**Fig. 2:**
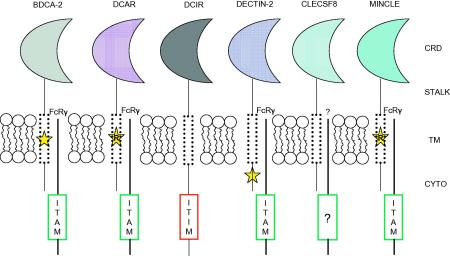
Cartoon representation of the Dectin-2 family of C-type lectins. Members of the Dectin-2 family of C-type lectins are type II proteins with a single C-terminal extracellular carbohydrate recognition domain (CRD), a stalk region, a transmembrane region (TM), and a cytoplasmic domain (cyto). DCIR contains an immunoreceptor tyrosine-based signalling motif (ITIM) in its cytoplasmic domain, while BDCA-2, DCAR, Dectin-2 and Mincle associate with FcRγ chain which contains an ITAM. It is not yet known whether Clecsf8 associates with an adaptor molecule (?). The star represents the region responsible for association with the adaptor. R, arginine.

The Dectin-2 cluster is comprised of Dectin-2, DCIR, DCAR, BDCA-2, Mincle and Clecsf8, which are members of the Group II C-type lectin family. They are all encoded by six exons and share a common structure, consisting of a single extracellular CRD, a stalk region of varying length, a transmembrane region, and a cytoplasmic domain which is generally short (with the exception of DCIR; Fig. 2). The receptors demonstrate type II transmembrane topology, where the C-terminal encodes the extracellular region of the protein and the N-terminal encodes the intracellular region. The presence of conserved residues typically associated with Ca^2+^ binding categorises these receptors as classical C-type lectins, and indeed Dectin-2 and Mincle have been shown to bind sugar ligands, but there are also non-carbohydrate ligands such as SAP130, an endogenous protein recognized by Mincle. Members of this family generally lack consensus signalling motifs in their cytoplasmic domains, however the presence of a positively charged residue in the transmembrane region of the receptors aids the association with ITAM-containing adaptor molecules, such as the FcRγ chain. DCIR is the only member of this family to contain a signalling motif (ITIM) and has been postulated to function as a paired immunoreceptor with other members of this cluster [23].

The Dectin-2 cluster has revealed exciting new insights into C-type lectin functions, although the physiological roles of most members remain poorly defined. The elucidated functions include fungal and viral pathogen recognition, sensing of necrotic cell death, facilitation of antigen cross-presentation, as well as control of the development of autoimmunity. In this review we will discuss each member of the family individually, describing expression, ligand recognition, signalling and known physiological functions.

### 2.1 Blood dendritic cell anitgen 2 (BDCA-2)

BDCA-2 is most well known as a specific marker for human plasmacytoid DCs (pDCs). BDCA-2 was originally identified by a monoclonal antibody panel raised against CD4^+^ blood DCs, and found to be exclusively expressed on CD11c^-^CD123^high^ pDCs with expression being lost upon maturation with IL-3 [24]. BDCA-2 transcript was weakly detected in tonsils, bone marrow, pancreas, testis, ovary, lymph nodes and peripheral blood leukocytes [25,26]. In tonsils, BDCA-2 protein expression was restricted to CD123^+^ pDCs found in the T cell-rich areas but not in the germinal centres [25]. Similarly, in immunohistochemical analysis of testis obtained from patients with testicular cancer, neoplastic and normal epithelium were negative for BDCA-2 staining while CD123^+^ pDCs were found to associate with lymphoid aggregates in tumours [25]. This suggested that expression of BDCA-2 in tissues was linked to infiltrating pDCs and it is now widely accepted that BDCA-2 expression is in fact restricted to pDCs. At least five truncated BDCA-2 mRNA species have been detected, which if translated would give rise to variants lacking different domains, including three variants lacking the transmembrane domain, which may represent soluble forms of the receptor [25,26]. To date, the murine homologue(s) of BDCA-2 remain unknown.

BDCA-2 contains an EPN motif in the CRD but ligand(s) for this receptor have not yet been identified. Furthermore, the ligands may not be mannose-based, as BDCA-2 from cell lysates was shown not to recognize these carbohydrates [26,27]. In the absence of a ligand, an anti-BDCA-2 monoclonal antibody was used to cross-link the receptor on pDCs and transfected U937 cells, resulting in src-family kinase dependent Ca^2+^ influx and intracellular protein phosphorylation [25,28]. Immunoprecipitation and expression studies in transfected 293T and Jurkat cells and freshly isolated pDCs, revealed that BDCA-2 couples with FcRγ chain [28,29]. Indeed, in Jurkat cells co-transfected with BDCA-2 and FcRγ chain, stimulation of BDCA-2 induced intracellular protein phosphorylation and Ca^2+^ influx which was not possible when BDCA-2 was expressed alone [25,28]. The association with FcRγ chain has been shown to take place through the transmembrane domain and this interaction is unusual as BDCA-2 does not contain a positively charged residue in this region [29].

Signalling through BDCA-2 is dependent on the ITAM motif in the FcRγ chain and downstream pathways involve Syk, Src homology 2 domain-containing leukocyte protein of 65 kDa (Slp65), Vav1, phospholipase C-gamma (PLCγ2) and Erk1/2 [28,29]. This cascade in primary human pDCs resembles signalling via the B cell receptor in B cells. BDCA-2 signalling does not however, lead to activation of the NF-κB pathway. In fact, cross-linking of BDCA-2 on pDCs actually decreased activation of the NF-κB pathway following stimulation with TNF-α [29]. Interestingly, amongst the proteins phosphorylated after BDCA-2 triggering, Röck et al. identified those involved in cytoskeletal rearrangement, including actin, tubulin and clatherin heavy chain [29]. This, and the fact that BDCA-2 contains an EEE (late endosomal sorting) motif in its cytoplasmic tail, may indicate targeting of the receptor to late endosomal compartments. Accordingly, when antibody was used to cross-link BDCA-2 on the surface of pDCs, BDCA-2-antibody complexes were internalised and efficiently presented to T cells, indicating a possible role for BDCA-2 in antigen capture and presentation [24,25].

Following BDCA-2 cross-linking on pDCs, the cells’ ability to produce IFN-α, IFN-β and IL-6 was found to be suppressed in response to CpG-DNA but the production of other cytokines, including IL-12p40, IL-12p70, IL-4 and IL-10, remained unaffected [25,28,29]. Additionally, when BDCA-2 was cross-linked on CpG-DNA stimulated pDCs, these pDCs had decreased ability to stimulate CD4^+^ T cells to proliferate and produce IFN-γ [30]. These stimulated CD4 ^+^ T cells also showed enhanced expression of CCR5 and decreased expression of CCR4, which are markers of Th2 and Th1 differentiation respectively [30]. These results indicate that BDCA-2 may function in skewing the immune system away from a proinflammatory Th1 response, by decreasing IFN production, towards a Th2 response, characterized by increased CCR5^+^ T cells. As pDCs have been shown to play a role in the pathogenesis of systemic lupus erythematosis, by being the main producers of increased levels of IFN-α/β associated with this autoimmune disease, BDCA-2 has been proposed to be a therapeutic target for treatment of this disease [31].

### 2.2 Dendritic cell immunoactivating receptor (DCAR)

There is only one publication on DCAR, a molecule identified by a cDNA homology search with the DCIR CRD [32]. DCAR transcripts were strongly detected in lung, spleen and bone-marrow DCs, weakly in skin and lymph node and not at all in bone-marrow NK cells [32]. The cytoplasmic domain of DCAR is characteristically short and lacks a defined signalling motif but the receptor was found to associate with the FcRγ chain, partly by association with an arginine residue in the transmembrane region of DCAR. In A20 cells co-transfected with the FcRγ chain, cross-linking a chimeric receptor consisting of the extracellular region of FcγRIIB coupled to the transmembrane and intracellular domains of DCAR, resulted in tyrosine phosphorylation of intracellular proteins and Ca^2+^ mobilization. When the tyrosine of the FcRγ chain was mutated to a phenylalanine, the Ca^2+^ mobilization was no longer observed, demonstrating that signalling from DCAR takes place via the ITAM motif of the adaptor. The FcRγ chain is also likely to be required for surface expression of DCAR, as it enhanced receptor expression in transduced in 293T cells. Two isoforms of DCAR have been identified, one of which lacks the stalk region, but the ligands and biological functions of these isoforms still remain undefined.

### 2.3 Dendritic cell immunoreceptor (DCIR)

DCIR was identified by screening a nucleotide database for molecules homologous to the Group II C-type lectin hepatic asialoglycoprotein receptors, which also contain a single CRD at the C-terminal end [33]. DCIR mRNA was found to be highly expressed in human peripheral blood leukocytes, and at lower levels in lymph node, spleen, bone marrow and thymus, while mouse DCIR was found to be expressed at highest levels in spleen and lymph node, although peripheral blood was not examined [33,34]. A closer look at protein expression on cells, revealed that DCIR was expressed on antigen presenting cells such as CD14^+^ monocytes, CD19^+^ B cells, macrophages, neutrophils as well as myeloid and plasmacytoid DCs (pDCs), but not on CD3^+^ T cells nor on CD56^+^ or CD16^+^NK cells [33–36]. *In vitro,* DCIR expression was found to be higher in CD14^+^ than CD1a^+^ derived DCs, while monocyte-derived DCs (moDCs) had high levels of expression throughout differentiation, which was downregulated upon maturation with LPS or CD40 ligand [33]. Additionally, DCIR expression on neutrophils was downregulated by TNF-α, IL-1α and LPS stimulation but anti-inflammatory stimuli, including IL-4, IL-10 and IL-13, did not affect expression, suggesting that DCIR may be downregulated during inflammation [35]. Interestingly, GM-CSF, IL-3, IL-4 and IL-13 stimulation of neutrophils resulted in accumulation of a short form of DCIR mRNA, which encodes a putative non-functional protein which may act as an antagonist to the full-length receptor [35,37].

DCIR is distinct from other members of this receptor family in that it possesses a longer cytoplasmic tail which contains a consensus signalling motif and is the first reported DC-expressed ITIM-bearing C-type lectin [33]. The presence of this ITIM motif, and downregulation of DCIR in proinflammatory settings, suggests a regulatory role for this receptor. The function of the intracellular domain of DCIR has been studied using B cells transformed with a chimeric receptor comprising the extracellular domain of FcγRIIB coupled to the intracellular domain of DCIR [34]. Stimulation of the B cell receptor in these cells resulted in Ca^2+^ mobilization and intracellular protein tyrosine phosphorylation and these activities could be inhibited by co-ligation with the chimeric receptor [34]. This inhibition was completely lost when the tyrosine of the DCIR ITIM motif was mutated to a phenylalanine [34]. Moreover, immunoprecipitations using a phosphorylated peptide covering the DCIR ITIM demonstrated association with the inhibitory phosphates, Src homology 2-containing tyrosine phosphatase 1 (SHP-1) and SHP-2 [38]. Accordingly, in pDCs, which are generally considered to induce Th1 polarization in response to viral stimuli, stimulation with TLR9 ligand resulted in the production of large amounts of IFN-α, which could be inhibited by cross-linking of DCIR with anti-DCIR antibodies, similar to BDCA-2 [36]. Cross-linking of DCIR on immature moDCs stimulated with other TLR ligands including LPS (TLR4), polyIC (TLR3), zymosan (TLR2 and 4) and R848 (TLR8), resulted only in the inhibition of the TLR8-mediated responses, demonstrating some selectivity in DCIR function [39]. Cross-linking DCIR in pDCs and moDCs also resulted in internalization of the receptor into intracellular vesicles in a clatherin-dependent manner [36,39]. Furthermore, once internalized, DCIR was able to deliver antigen into the antigen-presentation pathway, resulting in efficient T cell proliferation [36].

DCIR also plays a role in the capture and transmission of HIV-1 by DCs [40]. Decreasing DCIR surface expression on human IM-moDCs with small-interfering RNA, or blocking the receptor with antibodies, significantly reduced transfer of HIV-1 virions to autologous CD4^+^ T cells [40]. Additionally, Raji-CD4 cells transiently expressing DCIR were shown to have both increased binding to HIV-1 and enhanced virus production, following infection, compared to DCIR-negative cells. It has been proposed that DCIR interaction with HIV could allow the virus to gain access to nondegradative endosomal organelles and lead to fusion of viral and endosomal membranes, allowing productive infection of the cells [40]. To study association with HIV-1, a DCIR mutant which lacked the stalk region was created. Although the receptor was expressed on the surface of Raji-CD4 cells, it did not increase attachment or replication of virus particles, indicating a crucial role for the stalk region of DCIR in HIV-1 interaction, perhaps by extending the CRD from the cell surface and making it available for attachment [40].

In addition to acting as a PRR for HIV, DCIR has been shown to play a role in controlling autoimmune disease [13]. Aged DCIR deficient mice were found to spontaneously develop joint abnormalities, have elevated levels of autoantibodies and show higher levels of CD11c^+^ DCs and a proportional expansion of T cell populations in their lymph nodes [13]. Additionally, in response to collagen-induced arthritis, young DCIR deficient mice were found to develop a more severe disease than their wild type littermates, indicating a protective role for DCIR [13]. DCIR deficient mice also had elevated levels of IL-4, IL-10, IL-17 and IL-23 after type II collagen immunization, consistent with a negative regulatory role played by the receptor [13]. Additionally, bone-marrow cells from DCIR deficient mice differentiated into DCs and proliferated more efficiently than wildtype cells, due to an increased responsiveness to GM-CSF. DCIR therefore appears to be crucial in maintaining appropriate DC numbers to prevent development of autoimmunity in mice [13]. When studying DCIR expression in patients with rheumatoid arthritis, the receptor was found to be abundantly expressed in synovial biopsies but was not found in those of healthy controls [41]. In these biopsies, the receptor was expressed on numerous cell types and surprisingly also on CD56^+^ NK cells and CD4^+^ and CD8^+^ T cells [41]. DCIR^+^ T cells in the synovial fluid were activated, as well as much more abundant, than those found in peripheral blood [41]. The function of DCIR on T cells in this disease setting is as of yet undefined. Overall, these observations suggest DCIR has an essential role in maintaining homeostasis of the immune system by controlling DC expansion and the development of autoimmune disease.

### 2.4 Dendritic cell-associated C-type lectin-2 (Dectin-2)

Dectin-2 is the most well characterized member of this receptor family and was identified as an over-expressed transcript in a myeloid leukaemia mouse model and in macrophages, neutrophils and pleuripotent myeloid precursors [42]. Although originally proposed to be Langerhans cell specific, the use of a Dectin-2 specific monoclonal antibody has demonstrated that the receptor is expressed predominantly in tissue macrophages, some DCs and at a low level on Langerhans cells and peripheral blood monocytes, where expression levels could be transiently increased upon induction of inflammation [43]. The Dectin-2 promoter was also defined as a Langerhans cell specific regulatory element and, while numerous splice forms of Dectin-2 mRNA were shown to be highly expressed in a Langerhans cell-like skin-derived cell line compared to other cell lines, these transcript were also found to be abundant in spleen and thymus [44,45].

Dectin-2 was predicted to have mannose binding activity due to the presence of an EPN motif in the CRD [42]. Use of a soluble form of the CRD of Dectin-2 as a probe, revealed that the receptor could recognize zymosan and numerous pathogens including *Candida albicans, Saccharomyces cerevisiae, Mycobacterium tuberculosis, Microsporum audounii, Trichophyton rubrum Paracoccoides brasiliensis, Histoplasma capsulatum* and capsule-deficient *Cryptococcus neoformans.* Although the level of binding to these pathogens differed greatly, binding could always be inhibited by chelation of Ca^2+^ or competition with mannose [46,47]. Additionally, a glycan microarray showed that the receptor had specificity for high-mannose structures [46].

Interestingly, Dectin-2 may also have an endogenous ligand on CD4^+^CD25^+^ T cells and interaction of the two molecules may mediate UV-induced immunosuppression [48]. More specifically, injection of a soluble form of Dectin-2 *in vivo* inhibited UV-induced suppression of contact hypersensitivity, supposedly by inhibiting the interaction of endogenous Dectin-2 with its putative ligand on regulatory T cells [48]. Indeed, UV-B irradiation was shown to increase Dectin-2 expression in Langerhans cells of the skin at both mRNA and protein levels [49]. It is possible that Dectin-2 recognises an endogenous ligand that is not a carbohydrate, perhaps via an alternative binding site to that which recognises fungi, as has been reported for other C-type lectins, such as Dectin-1 [50].

Delivering antigens directly to DCs has the potential to increase the efficacy of vaccination and Dectin-2 has been proposed to act as a target for this delivery [51]. Carter et al. showed that even though Dectin-2 is expressed at low levels on DCs, anti-Dectin-2 monoclonal antibodies conjugated to ovalbumin were capable of targeting this model antigen to the cells and increasing presentation to CD8^+^ T cells [51]. Induction of a CD8^+^ T cell response could even be achieved with an antigen dose which was too low to induce a response when administered alone [51].

As mentioned, Dectin-2 has been identified as a PRR for fungi but the receptor appears to exhibit preferential recognition of hyphal over conidial forms [46,47]. Binding of *C. albicans* hyphae by RAW cells transduced with Dectin-2 resulted in tyrosine phosphorylation of intracellular proteins [47]. Cross-linking the receptor with a specific antibody demonstrated similar protein phosphorylation levels, as well as internalization of the receptor into endosomes, activation of transcription factor NF-κB and production of TNF-α and IL-1 receptor antagonist, indicating that the receptor is able to transduce intracellular signals [47]. Dectin-2 has the characteristic short cytoplasmic domain which lacks known signalling motifs and associates with FcRγ chain to transduce these signals [47]. Interestingly, it was not the positively charged arginine in the transmembrane region which was responsible for association with FcRγ chain, as has been shown for other receptors, but rather a short region of the cytoplasmic domain proximal to the transmembrane region [47].

Most recently, Dectin-2 has been shown to play a role in response to allergens [52]. Dectin-2 on bone-marrow-derived DCs (BMDCs) was able to bind to extracts from house dust mite (*Dermatophagoides farinae* and *Dermatophagoides pteronyssinus*) and *Aspergillus fumigatus* in a mannose-dependent manner [52]. Stimulation of mast cells co-expressing Dectin-2 and FcRγ chain with these extracts resulted in production of cysteinyl leukotrienes, proinflammatory lipid mediators which are not produced by untransfected cells [52]. Additionally, in primary BMDCs, signalling by Dectin-2 to produce cysteinyl leukotriene in response to the extracts was dependent on Syk kinase and FcRγ chain, and lentiviral knockdown of the receptor significantly reduced this activity [52].

Most of the work studying Dectin-2 has been performed in the mouse, however two reports describe identification and characterization of human Dectin-2 (hDectin-2) [53,54]. In tissues, hDectin-2 transcripts were detected in lung, spleen, lymph node, leukocytes, bone marrow and tonsils, but unlike mouse, Dectin-2 was not expressed in the human thymus. In peripheral blood cells, hDectin-2 transcripts were shown to be preferentially expressed in plasmacytoid, rather than myeloid, DCs and constitutively expressed in CD14^+^ monocytes and B cells, and could be induced in CD4^+^ T cells upon activation with Con A [53,54]. Indeed, similar to mouse Dectin-2, hDectin-2 appears to be upregulated in inflammatory settings, as gene expression in CD14^+^ monocytes could be upregulated by treatment with GM-CSF, TGF-β1 and TNF-α and downregulated with the addition of IL-4 and IL-10 [54]. In CD8^+^ T cells however, transcripts were detectable in inactive cells but decreased upon activation with phytohemagglutinin [54]. hDectin-2 was also expressed on Langerhans cells, however, while this expression was upregulated upon UV-B radiation in mice, it was downregulated in human cells [54]. These contradicting findings may be due to the fact that CD14^+^ monocytes were used as a surrogate model for epidermal Langerhans cells in the human experiment [49,54].

Interestingly a truncated isoform of hDectin-2 has been identified which lacks part of the intracellular domain and most of the transmembrane domain of the receptor [54]. The lack of transmembrane region has been proposed to encode a secreted protein which may act as an antagonist to full-length Dectin-2 [54]. Alternatively, this truncated version may act in a similar manner to human Dectin-1 isoform E, which also lacks a transmembrane region. This isoform was retained intracellularly where it interacted with Ran-binding protein, a molecule which is presumed to act as a scaffold protein to coordinate signals from cell surface receptors with intracellular signalling pathways [55].

### 2.5 Clecsf8

Murine Clecsf8 was first identified through a differential display PCR screen of numerous cell lines for macrophage-specific genes [56]. Northern blot analysis of mouse tissues revealed that Clecsf8 transcripts were predominantly expressed in resident peritoneal macrophages and at lower levels in bone marrow, spleen, lung and lymph nodes, however peripheral blood was not examined [56]. The Clecsf8 open reading frame encodes a 219 amino acid protein with typical Group II characteristics, including a short cytoplasmic domain with no signalling motif, and a CRD with conserved Ca^2+^ association residues [56]. The CRD however, does not contain the conserved EPN or QPD amino acid triplets associated with carbohydrate recognition and the transmembrane region lacks charged residues normally involved in association with an adaptor molecule. Cross-linking of Clecsf8 on 293T cells transiently expressing the receptor resulted in internalization of the molecule, indicating a potential role for the receptor in antigen uptake [57].

The human orthologue of mouse Clecsf8 was also found to be expressed in a monocyte/macrophage restricted manner, and interestingly, freshly isolated peripheral blood monocytes were found to have higher mRNA levels than cultured or buffy-coat isolated monocytes [57]. The expression of this receptor could be upregulated on these cells by stimulation with IL-6, IL-10, TNF-α or IFN-γ, but was downreglated with LPS [57]. Clecsf8 transcripts were detected in peritoneal macrophages from a patient suffering from *Pseudomonas aeruginosa* induced peritonitis as well as in synovial fluid macrophages isolated from a patient with rheumatoid arthritis, but not from a patient with gout [57]. In gout, macrophages have been implicated in the generation of anti-inflammatory rather than proinflammatory cytokines [58]. Although no ligand or biological function has as yet been described for Clecsf8, the receptor has been shown to be upregulated at the transcript level in a number of disease settings, including TNF-α over-expressing myocarditis and *M. tuberculosis* infection [59,60]. Overall these preliminary indications suggest that Clecsf8 could be upregulated in proinflammatory settings, as has been described for Mincle and Dectin-2.

Very little is known about the true function of the receptor although a Clecsf8-deficient mouse has been generated, offering an exciting reagent for future studies. This mouse has been generated and phenotypically characterized by The Consortium for Functional Glycomics (www.functionalglycomics.org/glycomics/publicdata/phenotyping.jsp). Clecsf8 deficiency suggested impaired T cell and B cell proliferation, following stimulation of the antigen receptors, as well as a possible increase in mature recirculating B cells found in the bone marrow, but none of these findings were significantly different to wildtype mice.

### 2.6 Mincle

The Mincle gene was originally identified as a transcriptional target of nuclear factor (NF-) IL-6 in peritoneal macrophages [61]. This transcription factor has low transcriptional activity unless activated by inflammatory stimuli and accordingly, Mincle gene expression can be induced in peritoneal macrophages upon stimulation with LPS, IFN-γ, IL-6 or TNF-α [61]. Mincle transcript was also detected in RAW macrophages and could be upregulated upon LPS stimulation, as well as LPS stimulated M1 myeloblastic leukaemia cells, but the receptor was not expressed in other cell lines including myeloma, mature B cells, NK cells, EL4 thymoma or NIH3T3 fibroblasts [61].

Like Dectin-2, Mincle was able to recognize fungi and induce inflammatory signals. Initially, microarray analysis of Mincle expression suggested that the gene was upregulated in bone-marrow-derived macrophages exposed to *Candida albicans* yeast and a soluble Mincle protein was subsequently found to bind to *C. albicans* and *Sacccharomyces cerevisiae* in an ELISA-based assay [62]. In this study, both human (in transduced cells) and mouse (in wildtype cells) Mincle were examined and it was found that although Mincle was not a phagocytic receptor for *C. albicans*, it was able to mediate inflammatory responses to the yeast. In transfected RAW cells, Mincle recognition of *C. albicans* induced the production of TNF-α, which could be partially inhibited by blocking with an anti-Mincle antibody and bone-marrow-derived macrophages from Mincle knockout mice produced less TNF-α than wildtype cells [62]. This study also used Mincle knockout mice to study systemic *C. albicans* infection and found that Mincle knockout mice had significantly higher fungal burdens in the kidneys than wildtype mice, demonstrating that the receptor was involved in pathogen clearance [62]. Interestingly, mice deficient in the NF-IL-6 transcription factor, which controls Mincle expression, are also susceptible to infection with *Candida* [63]. However, the susceptibility of knockout animals to a lethal dose of fungus has not been investigated.

Most recently however, another group were not able to demonstrate the recognition of *Candida* by Mincle. Using a non-myeloid cell-based NFAT-GFP reporter system to screen 50 different fungal species, including *Candida* spp. and *S. cerevisiae*, Mincle was found to recognize only *Malassezia* species [64]. It is however important to note that the *Candida* spp. screened in this system were not the same strains used in the study described above. As Mincle contains an EPN motif, it was postulated that it specifically recognises mannose on the fungal surface and accordingly, mutation of the EPN into QPD resulted in loss of recognition [64]. Additionally, a carbohydrate microarray showed soluble Mincle bound a multivalent form of α-mannose in a Ca^2+^ dependent manner [64]. Upon stimulation with *Malassezia*, bone-marrow-derived macrophages from wildtype mice showed increased Mincle expression and production of MIP-2, TNF-α, KC and IL-10, which was significantly reduced in Mincle knockout cells [64]. This indicates a role for Mincle in eliciting an immune response to *Malassezia* by macrophages. Additionally, intraperitoneal injection of *Malassezia* resulted in impaired IL-6 and TNF-α production as well as neutrophil infiltration in the Mincle deficient mice, compared to their wildtype littermates [64].

Similar to BDCA-2, DCAR and Dectin-2, Mincle has been shown to associate with FcRγ chain in transduced HEK293T cells and mouse peritoneal macrophages [65]. This interaction was found to take place via the positively charged arginine in the transmembrane region of Mincle and was crucial for signalling through the receptor [65]. Indeed, cross-linking of Mincle on thioglycollate elicited peritoneal macrophages with an anti-Mincle antibody resulted in the production of TNF-α, MIP-2, KC and IL-6, and this activity was lost in FcRγ deficient cells [65]. This signalling was shown to follow the Syk and caspase recruitment domain protein (CARD9) pathway and to be independent of MyD88 signalling, suggesting that Mincle does not require cooperation with TLRs to induce cytokine production [65]. While FcRγ chain was not essential for cell surface expression of Mincle, LPS stimulation was found to induce less Mincle expression on the surface of FcRγ deficient macrophages than wildtype cells, indicating that the adaptor was involved, at least partly, in surface expression of the receptor [65].

**Fig. 3:**
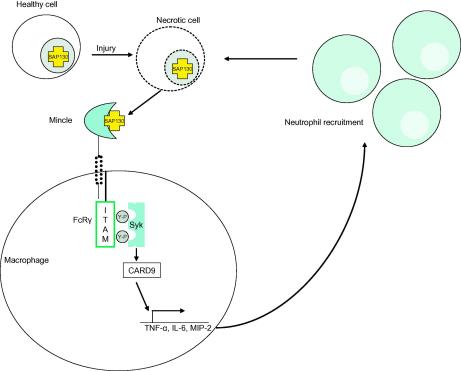
Mincle is a detector of necrotic cell death. Necrotic cell death induced by injury, such as radiation, results in the release of preformed nuclear proteins such as SAP130. SAP130 is recognized by Mincle expressed on the surface of macrophages, which signals via FcRγ chain, in a Syk and CARD9 dependant pathway. This induces the production of proinflammatory cytokines such as MIP-2, IL-6 and TNF-α, resulting in recruitment of neutrophils to the site of necrosis. (Adapted from: Brown GD. Sensing necrosis with Mincle. Nat Immunol 2008;9:1099–1100).

In addition to fungi, Yamasaki et al. have shown that Mincle can mediate inflammatory responses to necrotic cells [65]. In a search for an endogenous ligand using the NFAT-GFP reporter cell system, the authors discovered that cellular activation occurred following prolonged cultured and noticed that this activity correlated with the presence of dead cells [65]. Mutation of the EPN motif in the CRD did not abrogate cellular activation in response to dead cells, nor was binding by a soluble Mincle protein inhibited in the absence of Ca^2+^, suggesting that Mincle was recognising a non-carbohydrate endogenous ligand [65]. Immunoprecipitation from dead-cell lysates identified spliceosome-associated protein 130 (SAP130), a soluble protein that is localized in the nucleus of living cells and released during cellular necrosis. Stimulation of macrophages or Mincle expressing T cell hybridomas with purified SAP130 resulted in production of MIP-2 and IL-2 respectively, showing that SAP130 is a functional endogenous ligand for Mincle and that recognition of this protein could act as a signal for excessive cell death [65]. To study the function of Mincle in response to necrotic cell death *in vivo*, Yamasaki et al. inhibited Mincle function with blocking antibodies and found decreased neutrophil recruitment and cytokine production in response to cell death [65]. This model therefore proposes that Mincle recognises SAP130 released by necrotic cells which results in cytokine production by macrophages and subsequent neutrophil infiltration into the damaged tissue (Fig. 3). Speculatively, this infiltration could be either beneficial or detrimental to the host. The neutrophils may aid in clearance of apoptotic cells and promote repair or they may induce acute inflammation with a pathological outcome, such as in autoimmune diseases. Indeed, the Mincle gene complex has been reported to be associated with rheumatoid arthritis. Further studies in Mincle deficient mice are needed to understand the role of the receptor in autoimmunity [13,66,67].

## 3 Concluding remarks

The Dectin-2 family are multifunctional receptors, and have been shown to be important in both homeostasis and immunity. Many can act as PRRs and they recognize a range of pathogens, including fungi and viruses and probably others. Many also recognize endogenous ligands, highlighting the importance of this family in homeostasis. Indeed, the Dectin-2 locus has been identified as a susceptibility region associated with autoimmune disorders, including multiple sclerosis, systemic lupus erythematosus, rheumatoid arthritis and type I diabetes [68]. DCIR has been shown to be pivotal in preventing autoimmune arthritis in mice, and it is possible that Mincle recognition of necrotic cells may also lead to exacerbated inflammation and autoimmunity. With most of the receptors in the Dectin-2 cluster yet to be fully characterized, these first glimpses of the diverse repertoire of ligands and functions reveals an exciting area of future research.
